# Supplementary material for: From sequence to enzyme mechanism using multi-label machine learning
Source: BMC Bioinformatics. 2014 May 19;15:150. doi: 10.1186/1471-2105-15-150 (PMC4229970; doi:10.1186/1471-2105-15-150)
Supplement: Additional file 2 — Java code of ml2db. Additional file ml2db_code.tar.gz contains the Java source code to run the multi-label machine learning experiments and save the results to database. The code’s Javadoc is included. [file 1471-2105-15-150-S2.zip › additional file 2/ml2db/ml2db/doc/test/ml2db/dataharness/class-use/DataTableTest.html]

Uses of Class test.ml2db.dataharness.DataTableTest


JavaScript is disabled on your browser.


- Overview
- Package
- Class
- Use
- Tree
- Deprecated
- Index
- Help

- Prev
- Next

- Frames
- No Frames

- All Classes

## Uses of Class test.ml2db.dataharness.DataTableTest

- Packages that use DataTableTest

  | Package | Description |
  | --- | --- |
  |  |  |
  | --- | --- |
  | test.ml2db.dataharness |  |
- - ### Uses of DataTableTest in test.ml2db.dataharness

    Subclasses of DataTableTest in test.ml2db.dataharness

    | Modifier and Type | Class and Description |
    | --- | --- |
    |  |  |
    | --- | --- |
    | `class` | `DataTableFourTest` |
    | `class` | `DataTableOneTest` Basic data table containing 3 columns with: instances, attributes, classes. |
    | `class` | `DataTableThreeTest` |
    | `class` | `DataTableTwoTest` |

- Overview
- Package
- Class
- Use
- Tree
- Deprecated
- Index
- Help

- Prev
- Next

- Frames
- No Frames

- All Classes
